# Frequencies of Behavioral Problems Reported by Parents and Teachers of Hearing-Impaired Children With Cochlear Implants

**DOI:** 10.3389/fpsyg.2019.01591

**Published:** 2019-07-16

**Authors:** Merle Boerrigter, Anneke Vermeulen, Henri Marres, Emmanuel Mylanus, Margreet Langereis

**Affiliations:** ^1^Department of Otorhinolaryngology, Radboud University Medical Center, Nijmegen, Netherlands; ^2^Donders Institute for Brain, Cognition and Behaviour, Radboud University, Nijmegen, Netherlands

**Keywords:** cochlear implant, hearing loss, behavioral problems, education, speech perception, receptive vocabulary

## Abstract

**Background:** Internalizing and externalizing behavioral problems were frequently reported in profoundly hearing-impaired (HI) children with hearing aids. Due to the positive effect of cochlear implants (CIs) on hearing and language development, a positive effect on behavioral problems was expected. However, there is no consensus about the frequency of behavioral problems in CI children, and studies are often based on one informant with the risk of missing behavioral problems in other contexts.

**Aims:** The first aim of this study was to investigate the frequency of behavioral problems in children with CIs as compared to a hearing normative sample. The second aim was to measure the agreement between the parents’ and teachers’ rates on the behavioral problem scales. And the third aim was to investigate the relation between speech perception, language skills and the frequencies of reported behavioral problems.

**Methods:** Of 71 CI children, 51% were girls and 49% were boys, and the mean age was 8.6 (*SD* = 3.3). Behavior was reported by parents using the Child Behavior Checklist (CBCL) and by teachers using the Teacher Report Form (TRF). Frequencies of behavioral problems of CI children (6–16 years) were compared to a normative sample with the chi square test. Parent-teacher agreement was measured with the intraclass correlation coefficient (ICC 2,1). Next CI children were divided into four ability level categories regarding speech perception and language skills. Frequencies of behavioral problems were compared between the categories with the chi square test.

**Results:** Parents and teachers of CI children reported similar frequencies of behavioral problems to the normative sample. Fair to low parent-teacher agreements were found on the behavioral problem scales. A significantly higher frequency of behavioral problems was reported in children with low speech perception and receptive vocabulary at school.

**Conclusion:** Parents and teachers report similar frequencies of behavioral problems children with CIs compared to a hearing normative sample. Children with lower speech perception and language levels are more at risk of developing behavioral problems at school. Adequate speech perception and language levels are found to be protective factors for the development of behavior.

## Introduction

Prior to the application of cochlear implants (CIs), profoundly hearing-impaired (HI) children used to have limited to no auditory access to sound and spoken language. The perception of speech and interaction with the environment is required for communication and the development of spoken language (Kuhl and Rivera-Gaxiola, 2008; Kral and O’Donoghue, 2010). Language is known to support emotional self-regulation and social-cognitive competence. The lack of understanding of the auditory and linguistic refinements of social and emotional language, such as intonation, sarcasm, recognizing emotions and the ability to attribute mental states of other people, interferes with the understanding of people, culture, emotions, and social rules (Vaccari and Marschark, 1997; Calderon and Greenberg, 2003; Moeller, 2007). Therefore, profoundly HI children with limited or no access to spoken language are at risk for developing social and emotional problems (Barker et al., 2009; Gentilli and Holwell, 2011). Indeed, enormous percentages reaching approximately 30–50% of profoundly HI children fitted with or without hearing aids were reported to exhibit behavioral problems (Mitchell and Quittner, 1996; Vostanis et al., 1997; van Eldik et al., 2004; van Eldik, 2005; van Gent et al., 2007). Behavioral problems can be divided into externalizing and internalizing behavioral problems. Externalizing behavioral problems in HI children manifest in behavioral symptoms such as conduct problems, aggression and hyperactivity (Mitchell and Quittner, 1996; van Eldik et al., 2004; van Eldik, 2005; van Gent et al., 2007; Stevenson et al., 2010; Theunissen et al., 2014a; Stevenson et al., 2015). Internalizing behavioral problems manifest in emotional symptoms such as depression, anxiety, withdrawal behavior and thought and attention problems (van Eldik et al., 2004; van Eldik, 2005; van Gent et al., 2007; Barker et al., 2009; Stevenson et al., 2010; Theunissen et al., 2011, 2014a). The frequency of behavioral problems in profoundly HI children without CIs is disturbingly higher than in a Dutch normative sample of normal hearing peers, of whom 16% showed behavioral problems (Verhulst et al., 1996; van Eldik et al., 2004). Even more concerning is that only a small proportion of approximately 10% of HI children with emotional or behavioral problems were referred for professional help (van Gent et al., 2007). The guidance of profoundly HI children with behavioral problems therefore requires substantial effort from and skills of the parents and teachers. Especially for children in special educational settings for the deaf, these children show more behavioral problems than those in mainstream education (Theunissen et al., 2014b). Therefore, parents and teachers need more support and training to prevent or remediate emotional and behavioral problems in profoundly HI children (Calderon and Greenberg, 2003).

Currently, CIs can provide children with severe or profound deafness auditory access to sound and often levels of spoken language communication (Svirsky et al., 2004; Niparko et al., 2010; Fulcher et al., 2012; Boons et al., 2013; Geers and Nicholas, 2013). Especially early cochlear implantation before the age of 12 months provides profoundly HI children more abilities to develop sufficient speech recognition and perception to achieve age-appropriate language development (Colletti et al., 2011; Leigh et al., 2013; Dettman et al., 2016). Whereas it has been expected that improved language skills will decrease emotional and behavioral problems in profoundly HI children without CIs (Barker et al., 2009), it is hoped that, based on improved hearing and language development after cochlear implantation, a decrease in the frequency of behavioral problems is also observed in these children.

Indeed, several studies found that better language and communication skills were related to fewer behavioral problems in CI children (Wiefferink et al., 2012; Theunissen et al., 2014a; Netten et al., 2018). In addition, some studies found that children with CIs even show similar levels of behavioral problems to their normal-hearing peers (Khan et al., 2005; Huber and Kipman, 2011; Theunissen et al., 2014a, 2015; Huber et al., 2015). This finding reflects a major improvement for profoundly HI children since the introduction of CIs. However, some studies still report that children with CIs show more externalizing behavioral problems, internalizing behavioral problems and peer problems (Huber and Kipman, 2011; Chao et al., 2015; Huber et al., 2015). Despite much research on social and emotional development in CI children, there is still no consensus about the frequency of behavioral problems. These frequencies range from nine percent (Wong et al., 2017) to 20–30% (Huber and Kipman, 2011; Chao et al., 2015; Theunissen et al., 2015). Studies with young children (*M* = 5.1 years) with an early age of implantation (*M* = 16 months) (Wong et al., 2017) reported fewer behavioral problems than studies with an older test age (>11 years) and a later age of implantation (>3.8 years) (Huber and Kipman, 2011; Chao et al., 2015; Theunissen et al., 2015). However, in most studies, the frequencies of behavioral problems were not reported (Khan et al., 2005; Theunissen et al., 2011, 2014b; Huber et al., 2015; Park et al., 2016).

Behavioral problems are often determined by questionnaires. However, questionnaires are highly specific with regard to the context as well as the children’s behavior (Brown et al., 2006; De Los Reyes et al., 2009). Parental questionnaires, especially those completed by the parent with greater exposure and knowledge of the child’s behavior, seem to be representative of the child’s behavior at home (Langberg et al., 2010; Schroeder et al., 2010) but not at school. Therefore, information gathering about behavioral problems in children should be based on multi-informant information from different contexts (De Los Reyes et al., 2009; van der End et al., 2012). In the aforementioned studies on behavioral problems in children with CIs, almost all studies used parental questionnaires only or combined them with self-reported questionnaires for children. Only Huber and Kipman (2011) and Huber et al. (2015) used parental questionnaires combined with teacher questionnaires. Huber and Kipman (2011) found that teachers rated significantly more CI children with peer problems and clinical behavioral problems than did parents, with a parent-teacher agreement correlation of 0.40.

The first aim of this study is to investigate the frequency of behavioral problems in children with CIs. Next we want to compare these frequencies to that of a hearing normative sample. Additionally, agreement between the parents’ and teachers’ rates on the internalizing, externalizing and total behavioral problem scales will be measured. Finally, the relation between speech perception and language skills with the frequencies of reported behavioral problems will be investigated.

## Materials and Methods

### Participants

Participants were retrospectively included based on consecutive sampling as part of standard CI follow-up between June 2011 and June 2016. The inclusion criteria were as follows: (1) between 2.5 and 16 years old, (2) being able to participate in the speech perception test, (3) being able to participate in the receptive vocabulary test, and (4) parents and teachers both returned the behavioral questionnaires. All children with a bilateral unaided pure tone average of 90 dB or higher averaged over the frequencies of 1000, 2000, and 4000 Hz before cochlear implantation. The parents and teachers of 71 children both returned the behavioral questionnaires. Only these children were included in this study. Of 12 children, only the parents returned the questionnaires, and of 25 children, only the teachers returned the questionnaires. These children were excluded from this study.

The children attended one of three types of educational settings: mainstream education, education for profoundly HI/deaf children (Special HI), and education for children with other special needs (Special Other). Of the three children attending the Special Other educational setting, one child was diagnosed with ADHD and ASS, one child was diagnosed with cerebral palsy and one child was diagnosed with developmental disabilities due to Noonan syndrome.

Most of the other children with additional problems in mainstream or special settings were diagnosed with ADHD or ASS or had motor disabilities or learning problems. Descriptive statistics of the CI participant group are listed in Table 1.

**TABLE 1:** Descriptive statistics of the total CI group and the two age groups.

	**Total CI Group (*n* = 71)**		**Age group**		
			**1.5–5 (*n* = 21)**	**6–16 (*n* = 50)**	
			
	***n***	**%**	***n* (%)**	***n* (%)**	***p***

Gender					
Girl	36	51	10 (48)	26 (52)	0.80
Boy	35	49	11 (52)	24 (48)	
Age at implantation					
< 3 years old	55	77	21 (100)	34 (68)	0.002^*^
≥ 3 years old	16	23	0 (0)	16 (32)	
Etiology of hearing loss					
Congenital	60	84	17 (81)	43 (86)	0.12
Acquired	7	10	4 (19)	3 (6)	
Idiopathic	4	6	0 (0)	4 (8)	
Uni- or bilateral					
Unilateral	32	45	8 (38)	24 (48)	0.60
Bilateral	39	55	13 (62)	26 (52)	
Educational setting					
Mainstream	38	54	10 (48)	28 (56)	0.81
Special HI	30	42	10 (48)	20 (40)	
Special other	3	4	1 (4)	2 (4)	
Additional problems					
Yes	11	15	2 (9)	9 (18)	0.49
No	60	85	19 (91)	41 (81)	

	M (*SD*)	Range	M (*SD*)	M (*SD*)	*p*

Test age (years)	8.6 (3.3)	2.5–15.8	5.0 (0.8)	10.2 (2.7)	0.000^*^
Age at implantation (years)	2.2 (1.7)	0.6–10.6	1.2 (0.5)	2.7 (1.9)	0.001^*^
Duration of implant use (years)	6.4 (2.9)	1.9–13.3	3.8 (0.8)	7.5 (2.8)	0.000^*^

^*^p < 0.05.

The participants were divided in two groups based on age, 1.5–5 years and 6–16 years. Descriptive statistics of both groups are presented in Table 1. The frequencies of behavioral problems in CI participants in the range 6–16 years were compared to a Dutch normative sample of 1,417 children aged from 6 to 16 years, of whom 50% were male and 50% were female. Fifty-four percent of the normative sample were between 6 and 11 years old, and 46% were between 12 and 16 years old (Tick et al., 2007).

### Materials

#### Behavior Questionnaires for Parents and Teachers

The Dutch version of the Child Behavior Checklist (CBCL) and the Teacher Report Form for teachers (TRF) were used (Achenbach and Rescorla, 2000, 2010; Verhulst and Van der Ende, 2013). Children between 1.5 and 5 years old were assed with the CBCL/1½-5 and the caregiver-TRF (C-TRF). The children between 6 and 16 were assessed with the CBCL/6-18 and the TRF. These standardized and validated questionnaires comprise questions about a child’s emotional state and behavior. Response options are: 0 = not at all, 1 = a little or sometimes, 2 = clearly or often. Scores were tallied for the Internalizing, Externalizing and Total Behavior Problem broadband scales. The Internalizing Problem Scale covers anxiety and depressive problems, and the Externalizing Problem Scale covers aggressive and rule-breaking behavior. Raw scores were converted to T-scores after correcting for age and gender; next, the T-scores were classified into two categories: normal or deviant. A T-score ≥ 60 reflects a deviant score. A deviant behavior score indicates a significantly higher prevalence of dysfunctional behavior.

#### Auditory Speech Perception

Auditory speech perception was assessed using a standard Dutch open-set identification test for children aged up to 16 years old containing the consonant – vowel – consonant words of Bosman and Smoorenburg (1995). This test was carried out in a sound-treated booth by an audiologist. Stimuli were presented in the sound field at a presentation level of 65 dB SPL. Scores are expressed as a percentage of correctly recognized phonemes. Scores at or above 85% are considered good speech-recognition scores clinically by Hicks and Tharpe (2002) as well as in our clinic.

#### Receptive Vocabulary

Receptive vocabulary was assessed with the Peabody Picture Vocabulary Test-III-NL (PPVT) by Dunn and Dunn (2005). The PPVT is a standardized and validated test for persons aged from 2.3 to 90 years. A series of four pictures per page are presented to the child. The examiner states a word describing one of the pictures; then, the child has to point to the picture the word describes. The outcomes are expressed as a receptive vocabulary quotient. The average age-appropriate quotient is 100 (*SD* = 15).

### Procedure

After a technical inspection of the CI, speech perception assessments were carried out by an audiologist or audiologist assistant. Before or after the technical inspection and speech perception test, the receptive vocabulary test was conducted by a language and speech pathologist or speech therapist. Parents were requested to complete a parent questionnaire about behavior problems at home and were provided a teacher questionnaire with the request to give it to the child’s teacher. They received the questionnaire with standardized instruction information and prepaid return envelopes for both questionnaires. The time period for returning the questionnaires was as soon as possible but within 3 months of the CI follow-up date. The questionnaires were analyzed by a psychologist. Children and their parents received an evaluation report afterward. Data for this study were retrospectively collected by file study.

### Ethical Considerations

The study was carried out in accordance with the recommendations of the Committee on Research Involving Human Subjects of the Radboud University Medical Center. All parents of the participants gave written informed consent for the use of the patient’s file data in accordance with the Declaration of Helsinki.

### Statistical Analyses

First, we investigated the frequencies of clinical deviant behavioral problems in CI children on the CBCL and TRF Internalizing, Externalizing and Total Behavioral Problem Scales. Next the frequencies of CI children aged 6–16 with normal and deviant behavior reported by parents and teachers were compared to the frequencies of children classified with normal and deviant behavior reported by parents and teachers of the normal hearing Dutch normative sample (Tick et al., 2007) with the chi square test for goodness of fit (*p* < 0.05). The agreement between the parents’ and teachers’ rates on the internalizing, externalizing, and total behavioral problem scales was tested with the two-way random total agreement intraclass correlation coefficient (ICC 2,1) (*p* < 0.05). The cutoff scores of Cicchetti (1994) were used. An ICC level below 0.40 is classified as a low parent-teacher agreement; between 0.40 and 0.59, the parent-teacher agreement level is fair; between 0.60 and 0.74, the parent-teacher agreement level is good; and from 0.75 and higher the parent-teacher agreement is excellent.

To assess the effects of speech perception and receptive vocabulary on the number of reported behavioral problems, the CI children were divided into four ability level categories. These four ability level categories were based on clinical levels of speech perception and receptive vocabulary. The clinical level for adequate speech perception was set at a phoneme score of ≥85%, and that for adequate receptive vocabulary was set on a receptive vocabulary quotient of ≥85. CI children who obtained low speech perception and low receptive vocabulary scores were classified as ability category 1. Children who obtained adequate speech perception and low receptive vocabulary scores were classified as ability category 2. Children who obtained low speech perception and adequate receptive vocabulary were classified as ability category 3. Children who obtained an adequate speech perception and adequate receptive vocabulary score were classified as ability category 4. Descriptive statistics of the CI children in the four ability level categories are presented in Table 2. The chi-square test of contingencies (*p* < 0.05), with Cohen’s *w* as a measure of effect size, was used to assess the differences in the frequencies of behavioral problems between the ability level categories. Ability category 3 was excluded from the analyses due to the small sample size of four participants. *Post hoc* analyses were performed using the chi square test for goodness of fit (*p* < 0.01).

**TABLE 2:** Descriptive statistics of the four ability level categories.

	**Ability level category**				
	**1 (*n* = 9)**	**2 (*n* = 18)**	**3 (*n* = 4)**	**4 (*n* = 40)**	
	
	***n* (%)**	***n* (%)**	***n* (%)**	***n* (%)**	***p***

Gender					
Girl	2 (22)	7 (39)	3 (75)	24 (60)	0.10
Boy	7 (77)	11 (61)	1 (25)	16 (40)	
Age at implantation					
< 3 years old	9 (100)	14 (78)	0 (0)	32 (80)	0.001^*^
≥ 3 years old	0 (0)	4 (22)	4 (100)	8 (20)	
Etiology of hearing loss					
Congenital	9 (100)	15 (83)	2 (50)	34 (85)	0.44
Acquired	0 (0)	2 (11)	1 (25)	4 (10)	
Idiopathic	0 (0)	1 (6)	1 (25)	2 (5)	
Uni- or bilateral					
Unilateral	8 (89)	8 (44)	3 (75)	13 (32)	0.01^*^
Bilateral	1 (11)	10 (56)	1 (25)	27 (68)	
Educational setting					
Mainstream	1 (11)	6 (33)	1 (25)	30 (75)	0.001^*^
Special HI	6 (67)	11 (61)	3 (75)	10 (25)	
Special other	2 (22)	1 (6)	0 (0)	0 (0)	
Additional problems					
Yes	3 (33)	4 (22)	1 (25)	3 (8)	0.17
No	6 (67)	14 (78)	3 (75)	37 (92)	

	M (*SD*)	M (*SD*)	M (*SD*)	M (*SD*)	*p*

Test age (years)	6.7 (2.0)	9.2 (3.8)	11.8 (1.5)	8.5 (3.2)	0.06
Age at implantation (years)	1.7 (0.7)	2.5 (2.3)	4.3 (1.2)	2.0 (1.5)	0.07
Duration of implant use (years)	5.0 (1.9)	6.7 (3.7)	7.6 (1.9)	6.4 (2.8)	0.40

^*^p < 0.05.

All statistical analyses were performed using IBM SPSS Statistics 25.

## Results

### Behavioral Problems in CI Children Reported by Parents and Teachers

#### Frequencies of the Parents’ and Teachers’ Reported Clinical Behavioral Problems Compared to Normative Data

First, we investigated the frequencies of clinical deviant behavioral problems in CI children on the CBCL’s and TRF’s Internalizing, Externalizing, and Total Behavioral Problem Scales.

Frequencies of behavioral problems of the total CI group and the two age groups are presented in Table 3.

**TABLE 3:** Frequencies of deviant internalizing, externalizing and total behavioral problems in the total CI group, and in the two age groups.

**Scale**	**Informant**	**Total CI group %**	**1.5–5 year %**	**6–16 year %**
Internalizing problem scale	Parents	23	19	24
	Teachers	21	19	22
Externalizing problem scale	Parents	10	10	10
	Teachers	15	24	12
Total problem scale	Parents	14	10	16
	Teachers	18	33	12

The frequencies of reported behavioral problems in CI children aged 6–16, the normative sample aged 6–16 (Tick et al., 2007) and chi-square results are reported in Table 4. The chi-square test for goodness of fit shows that parents of CI children aged 6–16 report comparable rates of internalizing, externalizing and total behavioral problems to parents of the hearing normative sample. Also no differences were found among the internalizing, externalizing, and total behavioral problems reported by teachers of CI children compared with teachers of the hearing normative sample.

**TABLE 4:** Chi-square test for goodness of fit results and frequencies of deviant internalizing, externalizing and total behavioral problems in CI children and the dutch normative sample.

		**Observed**	**Expected**		
				
**Scale**	**Informant**	**CI group 6–16 year %**	**Dutch normative sample^a^ %**	**χ^2^ (1 df)**	***p***
Internalizing problem scale	Parents	24	27	0.23	0.63
	Teachers	22	18	0.54	0.46
Externalizing problem scale	Parents	10	21	3.65	0.06
	Teachers	12	23	3.42	0.07
Total problem scale	Parents	16	22	1.05	0.31
	Teachers	12	19	1.59	0.21

*^a^Tick et al. (2007).*

#### Relationship Between the Parents’ and Teachers’ Reported Context-Related Behavioral Problems in CI Children

**FIGURE 1:**
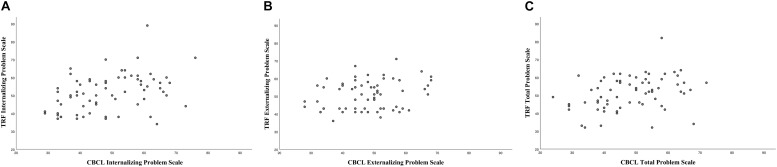
**(A–C)** Intraclass correlations between teachers’ (TRF) and parents’ (CBCL) ratings on the internalizing, externalizing, and total problem scales.

The results show overall fair to low agreements between the rates of reported behavioral problems by parents and teachers. The ICCs between the parents’ ratings on the CBCL Internalizing Problem Scale and teachers’ ratings on the TRF Internalizing Problem Scale were 0.40 (*p* = 0.000), those on the Externalizing Problem Scale were 0.28 (*p* < 0.01), and those on the Total Problem Scale were 0.32 (*p* < 0.01). Visual representations of the agreements between teachers’ and parents’ ratings on the three problem scales are shown in Figures 1A–C.

### The Effects of Speech Perception and Receptive Vocabulary on Reported Frequencies of Behavioral Problems

Table 5 shows the results of the total CI group in the four ability level categories on the speech perception and receptive vocabulary test.

**TABLE 5:** Speech perception and receptive vocabulary results of the CI children divided in four ability level categories.

		**Ability level**			
		**Low**	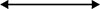		**Adequate**
		
		**1 (*n* = 9)**	**2 (*n* = 18)**	**3 (*n* = 4)**	**4 (*n* = 40)**
Speech perception					
	Mean (SD)	77 (7.9)	93 (5.1)	77 (9.3)	96 (3.6)
	Range	60–84	85–100	63–84	90–100
Receptive vocabulary					
	Mean (SD)	65 (9.6)	74 (9.9)	99 (14.2)	99 (10.1)
	Range	55–83	63–84	86–113	85–132

The results of the chi-square test of contingencies and the frequencies of reported internalizing, externalizing and total behavioral problems of CI children divided into the four ability level categories are presented in Table 6. No effect of ability level categories on the frequencies of internalizing and externalizing behavioral problems was found. An effect of ability level categories on the frequencies of behavioral problems reported by teachers was found only on the Total Problem Scale in the TRF. This effect had a Cohen’s *w* of 0.50, indicating there was a large effect of the ability level on the reported frequencies of total behavioral problems at school. *Post hoc* analysis showed that there were significant differences between the frequencies of reported behavioral problems at school in the lowest ability level (category 1) and ability level category 2, χ^2^ (1, *n* = 27) = 8.862, *p* < 0.01, and between ability level category 1 and the adequate ability level category 4, χ^2^ (1, *n* = 49) = 14.52, *p* < 0.001. No difference was found between categories 2 and 4.

**TABLE 6:** Chi-square test of contingencies results and frequencies of reported behavioral problems reported by parents and teachers of CI children divided into speech perception and receptive vocabulary ability levels.

		**Ability level**				
		**Low**	**⟷**	**Adequate**		
				
**Scale**	**Informant**	**1 (*n* = 9)**	**2 (*n* = 18)**	**4 (*n* = 40)**	**χ^2^**	***p***
Internalizing problem scale	Parents	22%	28%	18%	0.80	0.67
	Teacher	33%	6%	23%	3.57	0.17
Externalizing problem scale	Parents	11%	11%	10%	0.02	0.99
	Teacher	33%	11%	13%	2.79	0.25
Total problem scale	Parents	22%	17%	13%	0.61	0.74
	Teacher	67%	11%	10%	16.82	0.00^*^

^*^p < 0.05.

## Discussion

Regarding the first aim of this study, the results showed that parents and teachers report a similar proportion of behavioral problems (internalizing, externalizing, and total) in profoundly HI children with CI compared to a hearing normative sample. Considering the previously reported high frequency of behavioral problems in profoundly HI children without CIs in the literature (30 to 50%), the difference is remarkable. The previous frequency of behavioral problems in profoundly HI children without implants was 1.6 to 2.7 times higher than that of the profoundly HI children with CIs in the present study (Mitchell and Quittner, 1996; Vostanis et al., 1997; van Eldik et al., 2004; van Eldik, 2005; van Gent et al., 2007). This difference in reported frequencies of behavioral problems in HI children with and without CI is probably due to the improved speech perception, whereas profoundly HI children with early implantation of CIs nowadays achieve speech perception scores comparable to children with moderate HI wearing hearing aids (Leigh et al., 2016; Nekes, 2016). However, Theunissen et al. (2014b) found that children with CIs show not the same, but less behavioral problems than moderate to severe HI children with hearing aids. They also found no correlations between unaided/aided degree of hearing loss and behavioral problems. These findings indicate that not only the degree of hearing impairment but also other factors are involved in developing behavioral problems in HI children. The other factors could include communicative abilities and language development. This present study confirms that language is also associated with behavioral problems in HI children with CIs in the school environment; children with low speech perception and poor language abilities show higher frequencies of behavioral problems reported by teachers.

Additionally, the results show overall fair to low agreements between the rates of reported behavioral problems by parents and teachers. These low agreement between parents’ and teachers’ rates of behavioral problems is probably due to the interaction between the environment and the communicative abilities of these CI children, as studies show that children’s behavior could be influenced by factors like school environment and family dynamics (Hinshaw and Lee, 2003). This could be an explanation for the differences found, since the children in the home environment communicate mainly with their parents and siblings. Educational contexts require different social, emotional and communication skills, there is more interaction with others like teachers and peers, than the home environment does (Calderon and Greenberg, 2003). Interactions with peers are still difficult for some children with CIs according to Huber et al. (2015). The low agreements between parents’ and teachers’ ratings of behavioral problems in CI children in the present study endorses the statement that information gathering about behavioral problems in children should be based on multi-informant information in different contexts (De Los Reyes et al., 2009; van der End et al., 2012).

Finally, regarding the relationship between speech perception and language skills with the frequency of reported behavioral problems, we observed a difference in the frequencies of total behavioral problems reported by teachers based on the ability level of the CI children. As expected, more children with low speech perception scores and low receptive vocabulary scores show clinically deviant behavioral problems compared to CI children with higher speech perception scores or both higher speech perception scores and higher receptive vocabulary scores. This is in line with other research; whereas adequate language and communication skills are known to prevent behavioral problems and better language development will lead to less behavioral problems (Theunissen et al., 2014a; Netten et al., 2018). In our study teachers reported that 67% (*n* = 6) of CI children with poor speech perception abilities and poor language development showed behavioral problems against 22% (*n* = 2) of the parents. This discrepancy between parents and teachers frequency ratings of behavioral problems in CI children with low speech perception and language abilities, and the overall low agreement between parents and teacher ratings could be attributed to the communication and language problems these CI children experience in the school environment as mentioned before. In addition to that, as suggested by Netten et al. (2015), enhancing the communicative abilities of HI children is likely to improve their social- and emotional functioning and diminish behavioral problems in the school environment. These findings may be somewhat limited due to the small sample sizes, especially in the low ability level groups. There are also group differences concerning unilateral and bilateral CI and educational setting. More children in the lowest ability level category have unilateral CI and are attending special schools. This could be related to low speech perception and receptive vocabulary, however, that is something that we have not investigated. Therefore, caution must be applied interpreting the differences in behavioral problems between the ability level categories.

### Limitations

The first limitation of the current study is that we only used one receptive vocabulary test as a language measurement. A larger test battery of receptive and expressive language and communication development would be better to investigate the influence of communication problems in CI children on behavioral problems (Netten et al., 2015). Second, our study group and the Dutch normative sample differ in age range, therefore we could only compare the 6–16 group (*n* = 50) of our study group with the normative sample. A third limitation are the small sample sizes and group differences when measuring the effects of speech perception and receptive vocabulary on reported frequencies of behavioral problems. The small sample sizes could have influenced the statistical power of the analyses. The group differences concerning unilateral and bilateral CI and educational setting of the ability level categories could affect the outcomes in reported behavioral problems, whereas HI children in special educational settings are often more likely to have additional handicaps, lower socioeconomic status (SES) and less-communicative competences (Mitchell and Karchmer, 2011). This can be a factor in the development of behavioral problems. Therefore, caution must be applied interpreting the differences in behavioral problems between the ability level categories. The final limitation is the missing information of SES levels in our study group. Research shows that low SES is a consistent factor associated with behavioral problems (Letourneau et al., 2011). In our study it is unclear what influence SES has on results found.

## Conclusion

Parents and teachers report similar frequencies of internalizing, externalizing and total behavioral problems in profoundly HI children with CIs compared to a hearing normative sample. Children with lower speech perception and language levels are more at risk of developing behavioral problems at school. It is therefore important to use a multi-informant approach from different environments. Adequate speech perception and language levels are found to be protective factors for the development of behavior.

## Ethics Statement

The study was carried out in accordance with the recommendations of the Committee on Research Involving Human Subjects of the Radboud University Medical Center. All parents of the participants gave written informed consent for the use of the patient’s file data in accordance with the Declaration of Helsinki.

## Author Contributions

ML and AV formulated the research question. MB and AV collected the data. All authors provided contributions to the analysis and interpretation of the data. MB wrote the first draft of the manuscript. AV, HM, EM, and ML provided the critical revisions. All authors approved the final version of the manuscript for submission.

## Conflict of Interest Statement

The authors declare that the research was conducted in the absence of any commercial or financial relationships that could be construed as a potential conflict of interest.
